# Unusual imaging findings of pancreatobiliary‐type intraductal papillary mucinous neoplasm

**DOI:** 10.1002/jgh3.12453

**Published:** 2020-11-16

**Authors:** Akinobu Koiwai, Morihisa Hirota, Atsuko Takasu, Katsuya Endo, Takayuki Kogure, Takayoshi Meguro, Keigo Murakami, Kennichi Satoh

**Affiliations:** ^1^ Division of Gastroenterology Tohoku Medical and Pharmaceutical University Sendai Japan; ^2^ Division of Pathology Tohoku Medical and Pharmaceutical University Sendai Japan

**Keywords:** computed tomography, endoscopy, gastroenterology, imaging, magnetic resonance imaging, pancreatic cancer, pancreato‐biliary

## Abstract

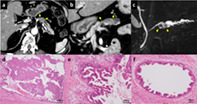

We experienced a rare case of main duct intraductal papillary mucinous neoplasm (MD‐IPMN) without overt mucin production. Histological findings classified the tumor as high‐grade dysplasia of pancreatobiliary‐type IPMN that has been reported to show high malignant potential with a property to disseminate extensively into pancreatic ducts.

## Introduction

Intraductal papillary mucinous neoplasm (IPMN) is a distinct type of pancreatic tumor characterized by mucin overproduction and potential progression to invasive carcinoma. IPMN can be morphologically classified into three types, that is, main duct (MD), branch duct (BD), and mixed types. MD‐IPMN is characterized by segmental or diffuse dilatation of the main pancreatic duct (MPD) without other causes of obstruction.^1^ We experienced a rare high‐grade dysplasia of MD‐IPMN without any characteristic imaging findings. Histologically, the tumor was classified as pancreatobiliary‐type IPMN and showed aggressive intraepithelial expansion into MPD and surrounding branch ducts.

## Case report and discussion

**Figure 1:**
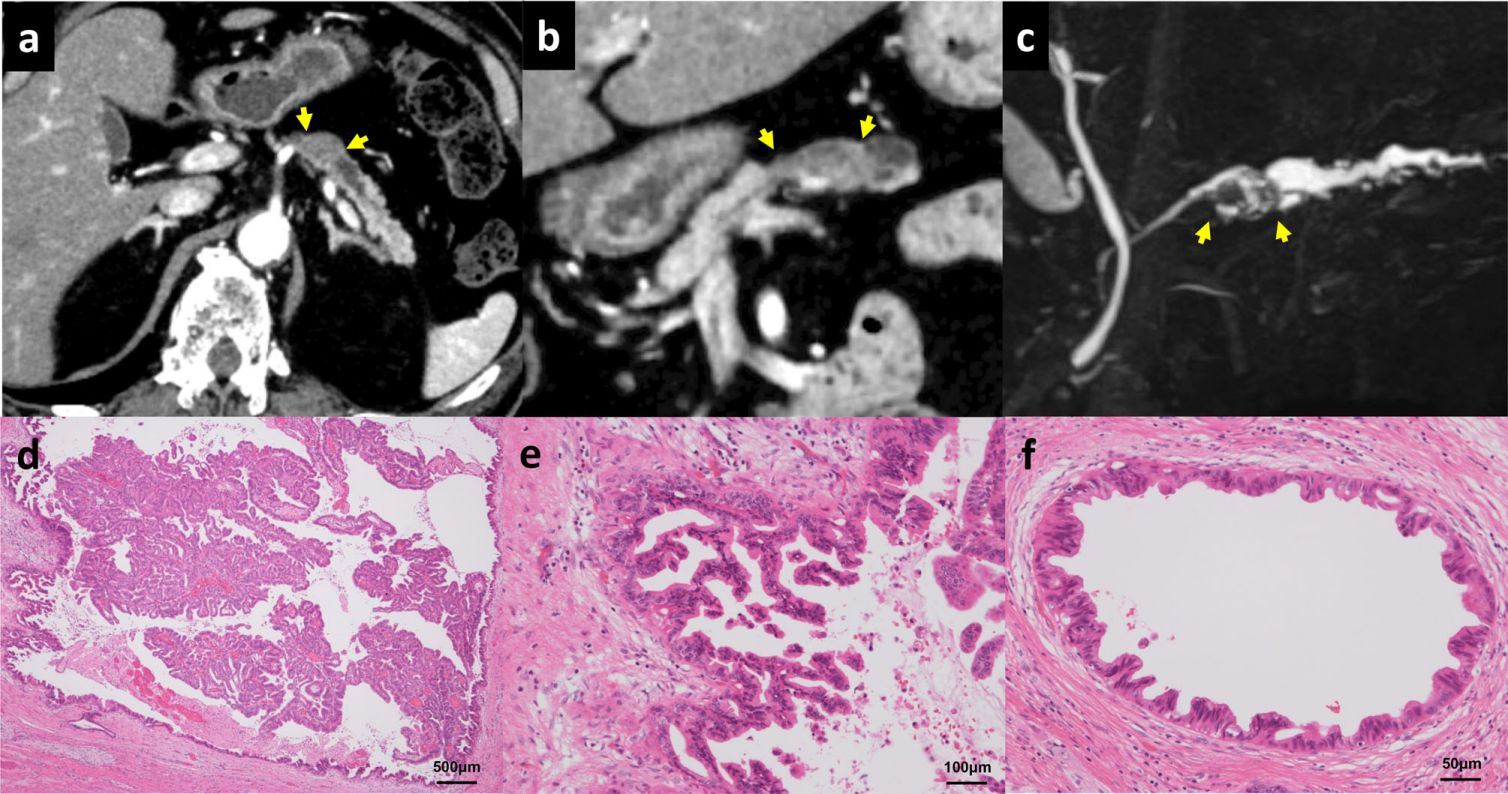
Imaging findings of the 15 mm‐diameter mass (between yellow arrows) in the body of the pancreas as shown by abdominal computed tomography in an axial image (a); a multiplanar reconstruction coronal image, which also shows the head of the pancreas having normal appearance (b); and MRCP (c). Hematoxylin and eosin staining showed an expanding papillary tumor in the MPD (×20) (d) and high‐grade dysplasia of the main duct epithelium (×100) (e), with spread into the branch ducts (×200) (f).

A 71‐year‐old man suspected of having pancreatic ductal adenocarcinoma (PDAC) was referred to our department. His laboratory tests revealed impaired glucose tolerance with an HbA1c of 10% and abnormally high values of two tumor markers, specifically CEA at 7.5 ng/mL and DUPAN‐2 at 260 U/mL. Serum amylase, liver function tests, CA19‐9, and SPAN‐1 were within normal limits. Contrast‐enhanced computed tomography (CE‐CT) showed a 15 mm‐diameter hypovascular tumor in the body of the pancreas with dilatation of the upstream MPD (Fig. 1a), while the head of the pancreas was normal, and the MPD was not dilated (Fig. 1b). Magnetic resonance cholangiopancreatography (MRCP) demonstrated a 15 mm‐diameter intraductal solid tumor in the dilated MPD (Fig. 1c). Endoscopic ultrasonography (EUS) showed a rounded, low‐echoic tumor with a homogenous internal structure and no infiltration of surrounding tissues. Duodenal endoscopy revealed no abnormalities of the major duodenal papilla and no fish‐mouth appearance. Endoscopic retrograde cholangiopancreatography (ERCP) showed an intraductal expanding tumor in the dilated MPD. However, neither a mucous plug nor downstream MPD dilatation was detected, both of which are expected findings in MD‐IPMN. The diagnostic workup, particularly CE‐CT, initially suggested PDAC. Subsequent MRCP, EUS, and ERCP findings indicated intraductal expanding tumor without overt mucin production, as opposed to invasive PDAC. Therefore, the differential diagnosis included acinar cell carcinoma, neuroendocrine neoplasm, and intraductal tubulopapillary neoplasm (ITPN). However, cytological analysis of pancreatic juice revealed an unexpected finding of high‐grade dysplasia of IPMN.

Although distal pancreatectomy was attempted, total pancreatectomy was ultimately required due to unexpectedly extensive intraepithelial tumor spread revealed by the intraoperative frozen section. Microscopically, the tumor had complex architecture of arborizing and interconnecting papillae without submucosal infiltration (Fig. 1d,e). The intraepithelial tumor spread was detected from the head to the tail of the MPD and extensively into the branch ducts of the body of the pancreas (Fig. 1f). Immunohistological analyses revealed that the tumor cells were positive for MUC1 and MUC5AC and were negative for MUC2. These findings classified the tumor as high‐grade dysplasia of pancreatobiliary‐type IPMN, a relatively rare subtype that has been reported to show high malignant potential with a property to disseminate extensively into pancreatic ducts, as well as a high risk of metachronous tumor development in the remnant pancreas.^2^, ^3^, ^4^ In most cases, these tumors are diagnosed after progression to invasive cancer.^5^ Therefore, high‐grade dysplasia of pancreatobiliary‐type IPMN, as demonstrated in this case, has rarely been reported.^6^
